# Barriers to the sustainability of an intervention designed to improve patient engagement within NHS mental health rehabilitation units: a qualitative study nested within a randomised controlled trial

**DOI:** 10.1186/s12888-015-0592-9

**Published:** 2015-09-02

**Authors:** Melanie Lean, Gerard Leavey, Helen Killaspy, Nicholas Green, Isobel Harrison, Sarah Cook, Thomas Craig, Frank Holloway, Maurice Arbuthnott, Michael King

**Affiliations:** 1 Division of Psychiatry, University College London, Maple House, 149 Tottenham Court Road, London, W1T 7NF UK; 2 The Bamford Centre for Mental Health and Wellbeing, University of Ulster, Magee Campus, Londonderry, BT48 7JL UK; 3 Rehabilitation Psychiatry, Division of Psychiatry, University College London, Maple House, 149 Tottenham Court Road, London, W1T 7NF UK; 4 Occupational Therapy, Centre for Health and Social Care Research, Faculty of Health and Wellbeing, Sheffield Hallam University, Montgomery House, 32 Collegiate Crescent, Collegiate Campus, Sheffield, S10 2BP UK; 5 Social Psychiatry, Health Service and Population Research Department, Institute of Psychiatry, King’s College London, London, SE5 8AZ UK; 6 South London and Maudsley NHS Foundation Trust, Maudsley Hospital, Denmark Hill, London, SE5 8AZ UK; 7 North London Service User Research Forum, Division of Psychiatry, University College London, Maple House, 149 Tottenham Court Road, London, W1T 7NF UK; 8 Primary Care Psychiatry and Joint-Director, UCL PRIMENT Clinical Trials Unit, Division of Psychiatry, University College London, Maple House, 149 Tottenham Court Road, London, W1T 7NF UK

## Abstract

**Background:**

We undertook a cluster randomised controlled trial to assess the effectiveness of a staff training intervention to improve patient engagement in activities in inpatient mental health rehabilitation units. Concurrently, we undertook a qualitative study to investigate the experiences of staff within the intervention units and the contextual issues that may have influenced the effectiveness of the intervention.

**Method:**

We conducted focus groups with staff working in the inpatient units that received the intervention, sampled using a maximum variation strategy.

**Results:**

The intervention was accepted by staff. However, the skills gained, and changes to the unit’s processes and structures that were agreed with the intervention team were not sustained after they left. The main reasons for this were a) external factors (economic recession, resource limitations); b) organisation level factors (lack of senior staff support; competing priorities); c) limitations of the intervention itself (length of intensive training period; reinforcement of skills).

**Conclusion:**

This study illustrates some of the inter-related factors which operate at different levels within and outside of NHS organisations that may impact on the success of complex interventions. These factors need to be considered when designing interventions to ensure adequate buy-in from senior staff.

**Trial registration:**

Current Controlled Trials ISRCTN25898179 (Registered 23 April 2010)

## Background

Mental health rehabilitation services provide specialist, tertiary care to people with complex problems who have not recovered adequately from an acute episode of illness to return home. Modern approaches to rehabilitation within mental health services emphasise the promotion of social inclusion, independence and autonomy using a whole system approach [1], regarded as crucial to successful placement in supported accommodation [2]. However, evidence from UK studies suggests that patients in inpatient mental health units are poorly engaged in the therapeutic activities [3, 4] that could facilitate this.

We undertook a cluster randomised controlled trial (RCT) to assess the effectiveness of a staff training intervention that aimed to improve patient engagement in activities in inpatient mental health rehabilitation units [5]. The intervention (called “GetREAL”), was based on theory and practice from occupational therapy and organisational psychology, and aimed to equip nurses and other unit staff with the confidence and skills to facilitate patient engagement in activities on the unit and in the community. Forty inpatient rehabilitation units across England were included in the RCT: 20 units were randomly allocated to receive the “GetREAL” intervention and 20 units to continue with standard care. The intervention described in detail elsewhere [5, 6], entailed a staged programme, with pre-disposing, enabling and reinforcing stages delivered by one of two small teams (the “GetREAL” intervention teams) comprising a senior occupational therapist, activity worker and a patient consultant who helped deliver the two training workshops. All units involved in the treatment arm of the trial received the full “GetREAL” intervention as detailed below.

During the predisposing stage, we aimed to gain support for the GetREAL intervention from senior unit managers and clinicians through a consultation meeting at each of the participating sites facilitated by a senior psychiatrist from the research team (HK, FH, and TC). The Enabling stage commenced with a training workshop which involved teaching and demonstrating motivational techniques and occupational therapy techniques [6] to staff that could be used to encourage service users’ engagement in activities. Following the training day, the occupational therapist and activity worker worked daily in the unit alongside staff for the rest of the five weeks to model and give intensive, hands on support for staff to gain confidence in the implementation of the techniques and interventions learned during the training day. During the enabling stage, the team also worked with staff to address barriers to change through the formulation of team-level action plans. The Reinforcing stage started during the fifth and final week of the intervention, when the GetREAL team facilitated a half day workshop. This workshop aimed to review the intervention with the service manager and staff to agree how the skills acquired and lessons learned could be incorporated into the unit’s routine practices and processes. A Link Person in each unit receiving the intervention was identified from the staff team to lead on the activity based action plan developed during the Enabling Stage of the intervention and to liaise with the intervention team over subsequent months. Despite the considered planning and delivery of this intervention the RCT found it to be less effective than anticipated. While there was an increase in patient engagement in activities seen at around half of the interventions sites, this was not significant when compared to the control sites [5].

## Method

We undertook this qualitative study alongside the RCT with the aim of investigating the experiences of staff within the intervention units and contextual issues that may have influenced the effectiveness of the intervention. This study comprises one component of the Rehabilitation Effectiveness and Activities for Life (REAL) research programme, funded by the National Institute of Health Research. The research was approved by the South East Research Ethics Committee (Ref. 09/H1102/45) and registered with Current Controlled Trials (Ref ISRCTN25898179) http://www.controlled-trials.com/ISRCTN25898179.

### Setting

We anticipated that a purposive sample of 10 rehabilitation units, which represented 50 % of all units that had received and implemented the GetREAL staff training intervention, would provide a spread of setting and staff experiences. Thus, we used a maximum variation strategy to include unit characteristics such as location of the unit (urban, suburban or rural), inpatient setting (within or on the grounds of a hospital; or community based inpatient units located in residential areas), unit size (number of beds), time post intervention and the GetREAL intervention team that delivered the intervention. All focus groups were conducted on the inpatient rehabilitation unit premises. We held the focus groups in a quiet setting, typically in a meeting or common room on the unit.

### Participant selection

All staff of the selected units were invited to participate in the focus group interviews. Attendance at focus groups was facilitated by the unit managers. No further purposive sampling of attendees was undertaken - we did not aim for “representativeness” of staff in these groups due to the logistic challenges that this would have involved given the constraints on staff and researcher time.

### Data collection

We chose focus group interviews as a means of efficiently generating multiple staff perspectives about the delivery of the GetREAL intervention and also to uncover some of the staff relationships and dynamics that affect the delivery of services. The pilot and initial interviews were facilitated by our qualitative lead (GL) and a co-facilitator (HK), with other researchers (ML/HG) observing as part of their training in focus group facilitation. Later interviews were facilitated by ML and co-facilitated by HG. At the beginning of each focus group, the facilitator explained to participants that the purpose of the focus group was to gain their feedback and thoughts on their experience of receiving and participating in the GetREAL intervention. Participants were assured as to the confidentiality of the interviews and that recordings (and subsequent transcripts) would be fully anonymised. It was made clear that the researchers worked separately to the intervention team and that we welcomed a frank discussion about their experiences.

A topic guide was developed with input from the Project Management Group (HK, GL, ML, SC, FH, TC, MK, NG, IH, MA) and consultation with the local Service User Research Forum. After piloting in one unit, the topic guide was reviewed and minimal changes were made to the structure and order of the questions. This pilot interview was considered sufficiently similar to subsequent interviews and was included in the final analysis. The topic guide covered the following areas: (a) the anticipation and preparation for the intervention team’s arrival; (b) understanding of the intervention’s structure and components; (c) the qualities of the intervention team and how they interacted within the unit: (d) benefits and deficits of the intervention; (e) changes in unit policy and practice; (f) maintaining the intervention. The facilitator was free to prompt or probe at their discretion to facilitate discussion amongst focus group members. Focus groups lasted on average 1 hour (range 30–90 mins). All staff provided written informed consent prior to participating.

All interviews were digitally recorded and transcribed verbatim (ML). Field notes were made during and after the focus group interviews. These notes served to capture any dynamics between staff or factors affecting the interviews that may not have been evident when reviewing the audio recording. Reflective notes were also taken during transcription. Emerging ideas and concepts were discussed with other members of the research team. In addition to the topic guide, these notes informed the preliminary coding frame.

### Data analysis

Guided by the aims of the study, which were reflected in the topic guide, we adopted a critical realist stance to carry out a thematic data analysis [7]. Thus, we intended documenting attitudes and behaviours rather than interpreting participants’ accounts. A sample of the transcripts were read and re-read and then coded separately by ML and GL. Fieldnote observations and theoretical memos were added to give explanatory value to the coding and the relationship between codes. Following this process, the coding was discussed with the wider research team who then agreed the thematic framework (see Appendix). All transcribed texts were then entered into a software programme for qualitative data (Atlas-ti.7) and systematically coded (ML). Coding began by exploring the broader themes in each transcript, which could be divided into two groups: those relating specifically to participant’s/staff views on participation in the GetREAL intervention; and those related to the provision of patient activity on inpatient rehabilitation units in general. From these two broad themes, transcripts were further analysed and categorised into sub-themes. Using a balance of both inductive and deductive analysis, we proceeded to examine the parallels, distinctions and patterns both within and between groups, which formed our analysis, and the findings we derived from these.

## Results

Focus groups were conducted with staff of 10 units between two and nine months (mean six months) after the GetREAL team had completed the Enabling Stage of the intervention and left the unit. These units had between 9 and 31 beds (mean 19 beds), half were community based and half were hospital units. Four were located in the inner city, three in suburban and three in rural areas. Five units had received the intervention from one GetREAL team and five from the other GetREAL team. The focus groups had a mean attendance of six staff members (range 2 to 14 participants) with a total 59 staff participating (3 unit managers, 25 nurses, 19 support workers, 7 occupational therapists, 1 clinical psychologist, 1 food technician, 1 activity worker, 1 student nurse and 1 consultant psychiatrist). More details are shown in Table 1. Although the groups comprised staff from different disciplines, we make no attempt here to provide detailed perspectives on each but rather to report the salient issues derived from the analysis. While sites and staff groups differed in terms of locale and resources, we found no striking distinctions between sites in terms of current concerns and challenges to the implementation of the GetREAL intervention.

**Table 1:** Unit and focus group characteristics

Unit ID	Date of Focus group	Time post intervention (months)	Intervention Team	Unit Size (Total Beds)	Location	Unit Type	Facilitator	Staff per focus group
0902	23/08/2011	5	2	20	suburban	community	GL/HK*	5
0502	24/08/2011	3	1	9	suburban	community	GL/HK*	9
2902	25/08/2011	2	2	31	rural	community	GL/ML	14
0102	20/09/2011	4	2	14	urban	hospital	ML/HG	7
0804	26/03/2012	7	1	25	urban	hospital	ML/HG	5
3301	08/11/2012	9	2	25	urban	community	ML	6
4203	05/12/2012	9	1	15	suburban	hospital	ML	2
3704	31/01/2013	9	1	20	urban	hospital	ML	4
4204	06/12/2012	7	2	15	suburban	hospital	ML	4
3106	21/02/2013	8	1	18	rural	community	ML	3
	Average	6		19			Total	59

*ML/HG observing

### The challenges of working in rehabilitation units

The focus group interviews allowed staff to reflect on the nature of rehabilitation units prior to the intervention. Predominantly, we note systemic problems that caused frustration among rehabilitation unit staff. Staff emphasised: (1) the complex nature of the client group predominantly consisting of those with a diagnosis of schizophrenia, complicated by non-response to first line drugs, cognitive impairment, negative symptoms, and comorbid drug and alcohol use, and which results in major impairments in social and everyday functioning; (2) impact of inappropriate referrals. For instance pressure on beds mean that patients are often prematurely transferred from acute settings with more active features of psychosis hence making engagement in rehabilitation challenging or due to the move of care into the community, those now referred to rehabilitation often have longer term needs that is not conducive to the move on ethos of rehabilitation services; (3) inadequate rehabilitation-focused leadership and staffing. These issues appeared to hamper staff in identifying and delivering a rehabilitative focus.

*People*’*s mental states on an inpatient unit are very up and down*, *so you could have a breakthrough one week*, *and then the following week the same service user is back to square one again*, *so it*’*s hard. And I suppose you*, *after time*, *you do start feeling de*-*motivated yourself*- *you feed off one another*” (*Occupational Therapist*: *Unit 0902*)

[*The unit*] *was bad at looking at people as individuals. It was like well this is how we*’*ve done it and we*’*ve done this for years and yeah we ain*’*t gonna change*”. (*Occupational Therapist*: *Unit 2902*)

### Impact of the GetREAL intervention

The receptivity to change by the staff teams was apparent across all focus groups. Reinforcing their views about the units prior to the arrival of the intervention, participants discussed their “*sense of optimism*” and wanting “*a different approach to thinking*, *to working and to patients*”. We identified five key themes in our analysis of how the intervention appeared to initiate change. These were: (1) Planning and reflection; (2) External and alternative perspectives; (3) Dissolving role boundaries; (4) Collective working and responsibility; (5) Motivation. The dynamics of change reflected in these themes should be regarded as interdependent; all contributed to an increase in staff morale and engagement.

#### 1. Planning and reflection

The intervention provided an opportunity for staff members to plan and reflect on the state of their service provision, to have their voice heard and feel valued. While successfully promoting the importance of structure and consistency to processes, the intervention also facilitated a more flexible approach to unit activities. For example, staff moved from strictly timetabled events to a patient-centred approach that was ad-hoc and needs based.

*It had an enormous benefit in that it were joint working and collaborative working and that it was going to bring everyone together*, *as one team*, *working in one direction*” (*Occupational Therapy Instructor*: *Unit 2902*)

*More flexible now*, *whereas before we were kind of more rigid you know so rather than thinking oh no we have to wait until this time and that day to do* [*activities*]”. (*Staff Nurse*: *Unit 0102*).

#### 2. External and alternative perspectives

Staff now critically examined taken-for-granted and traditional practices. This helped staff to use a more individualised approach to meeting patients’ needs that facilitated patients to make choices and increased their confidence.

*The morning shift had to get everybody*’*s laundry done in that shift*…*sort of at our convenience. Why does it have to be like that*? *Why can*’*t we be a bit more flexible*? *And it was just having some fresh eyes coming in and seeing* (*Occupational Therapist*: *Unit 2902*)

*Some patients are harder to engage*, *but*…*now I just try harder to find something that they like*, *that will kind of motivate them*, *personally*, *like more individually rather than as a whole*, *trying the same thing for everybody*, *but now I try to really pick something that will get them like* “*ah ok*” (*Support Worker*: *Unit 3704*).

#### 3. Dissolving role boundaries

The GetREAL team played an important role in providing permission for staff to move beyond the confines of their perceived role remit. A rigidly hierarchical staffing culture had inhibited therapeutic engagement with patients.

*For a lot of healthcare assistants*, *there*’*s issues of power and who actually gives permission*… *and I think*, *depending on how the structure works*, *the hierarchy works*, *it perhaps is hard for some people to feel* ‘*actually I think it would be really good to do swimming*, *to do whatever. But am I*? *Is that alright on this shift*?” (*Staff Nurse*: *Unit 2902*).

Increased confidence and a sense of agency may account for the shift in unit atmosphere from apathetic and stagnant to one of enthusiasm and possibility that was reported by staff across several units.

[*Activity workers*] *particularly benefited from the developing role and greater sense of confidence and I think with that has also come much greater initiative in terms of setting up certain activities and ways of working with our patients*. (*Consultant Psychiatrist*: *Unit 0804*)

#### 4. Collective working and responsibility

Staff acknowledged that the intervention reinforced that engaging patients in activity was the responsibility of every staff member. This encouraged them to work better as a team, increasing responsibility and accountability.

*It just reminds everybody that it*’*s an MDT* (*multi*-*disciplinary team*) *responsibility*, *not just the OT*’*s* (*Charge Nurse*: *Unit 4203*)

*Prior to the GetREAL team coming*, *the occupational therapy staff tended to run those groups and I think there was perhaps a view from the nursing staff that if they were doing a group it was almost seen as an add*-*on extra. And I think what the GetREAL team was trying to say was* ‘*no it*’*s not an add*-*on extra*, *this is part of everybody*’*s role*’” (*Occupational Therapy Instructor*; *Unit 2902*).

#### 5. Motivation

The GetREAL intervention motivated staff towards meaningful engagement with patients. This quote below illustrates the dialectical relationship between staff and patients. Thus, staff described the intervention in terms of empowering and motivating both staff and patients.

*There was a renewed enthusiasm*… *it did make people think I could do something and I could start it and did*. (*Staff Nurse*: *Unit 3106*).

*We see them [the patients] happy as well*, *makes us happy if we see them engaged*, *yes it gives us satisfaction*. (*Healthcare Worker*: *Unit 0902*)

### Barriers to sustainability

Despite the apparent staff enthusiasm for the GetREAL intervention and its ability to transform organisational processes and practices, staff morale and patient engagement, our trial did not demonstrate better outcomes in the GetREAL intervention arm [5]. Analysis of the staff focus groups indicated the perceived barriers to sustained change and benefit. We used the definition of sustainability provided by Shediac-Rizkallah and Bone [8], in which the key aspects of program sustainability are defined as 1) maintenance of health benefits from the program; 2) institutionalization of a program within an organization; and 3) capacity building in the recipient community. An overarching explanation was that staff appeared to revert to previous demarcations and behaviours. The following explanatory factors can be divided into those that were intervention-related (1–3) and contextual issues (4–7): (1) insufficient preparation; (2) brevity of engagement with the intervention team; (3) misunderstanding of the aims of the intervention; (4) resources; (5) role boundaries; (6) leadership; (7) competing priorities.

#### Insufficient preparation

We noted that some units felt insufficiently prepared for the intervention with some staff receiving minimal information prior to the GetREAL team’s arrival on the unit. As part of the RCT, unit managers were instructed to minimise the information they gave staff in order to prevent unmasking of the researchers to the unit’s allocation as an intervention or comparison unit. The researchers collected baseline data at each unit involved in Phase 3 within the four weeks prior to the GetREAL team’s arrival which meant that there was only one or two weeks available for the unit staff to prepare for the arrival of the GetREAL team. This may have affected staff readiness and receptiveness to change.

*It did seem rather rushed. We didn*’*t get time to really explore it as a team or discuss things* ‘*cause as you say*, *you all came one day and then bang it had started*. (*Unit Manager*: *Unit 0502*)

*I think there was a bit of confusion*, *because of the randomised controlled trial and us being told not to say things about who*’*s having an intervention*, *that was a bit confusing when one of the researchers were here*. (*Staff Nurse*: *Unit 2902*)

#### Brevity of engagement with the intervention team

Staff felt that the length of the intervention meant that its aims were unrealistic and that the short duration of the intervention had consequences for its implementation.

*There was lots of ideas and I think for the five weeks*, *to get those ideas up and running*, *I think that was a bit of a stumbling block really*… *and now you*’*ve got your lack of manpower, you*’*ve got your lack of this*, *the ward itself and the type of clientele we have*… (*Unit Manager*, *Unit 0102*)

*Perhaps if they were here longer. We need to implement a lot of the stuff* … *it didn*’*t give them that much time to implement their ideas*. (*Nursing Assistant*: *Unit 3301*)

#### Misunderstanding of the aims of the intervention

Also noted, was some confusion over the aims of the GetREAL intervention with some staff appearing to think that the intervention was targeted at patients, not the staff themselves. For example, one nurse quoted below, spoke of patients who did not have the involvement of the GetREAL team because they were admitted after the intervention had been delivered, and the team had left. Thus, it appears that some staff misunderstood that the intervention was aimed at creating enduring change in practice that would in turn benefit patients, rather than patients benefiting directly from the GetREAL team’s input.

*Well the client group changes as well. So probably since they last came here*, *we*’*ve had 8 new people in*, *who have not had that involvement from the GetREAL service*. (*Staff Nurse*: *Unit 3106*)

It was also suggested that the patients present on units during the GetREAL intervention were somehow not representative of their typical patients and hence were easier to engage:

*When they* [*the GetREAL team*] *came*, *they were dropped into a golden opportunity when we did have a fair few clients that you could motivate*. (Unit Manager: *Unit 0502*)

#### Resources

Staff commonly cited inadequate resources to explain the inconsistent delivery of activities. As the quote below indicates, unit staff appeared anxious that their time must be justified and measurable. Paperwork and office based tasks tended to be prioritised over less tangible outcomes such as patient engagement. This is potentially reflective of the economic pressures on services at the time (see Discussion section). While we designed the intervention activities to be conducted within the constraints of current staff numbers, it is possible that the planning and coordination of activities performed by the intervention team, as part of the modelling exercise, could not be sustained in their absence.

*Well I don*’*t particularly enjoy it*, *I*’*d soon be with patients than sat filling forms in myself*, *but it just justifies a moment in time*, *with all the cutbacks in all this country*, *you*’*ve got to justify what you*’*re doing*, *and it just makes you feel better that it*’*s another job you*’*re doing*”. (*Staff Nurse*: *Unit 0502*)

*I think we still lack a good strong skill base for that very*, *very basic motivating work with clients*…*there are staff on the team that are very good at that and others who just don*’*t seem to*.. *And unfortunately I think it*’*s a slightly attitudinal thing. That it*’*s not the most important part of their job really*. (*Unit Manager*: *Unit 3704*)

#### Role boundaries

While the intervention assisted in challenging staff views about rigid role specifications, encouraging nurses and support workers to facilitate activities, this was not sustained once the GetREAL intervention team had left. Many staff appeared to revert to a view that activities were not part of their responsibility. This is particularly relevant since the staff typically responsible for providing directives on a unit - nurses and unit mangers - appeared to disregard therapeutic activity as a priority

*If we nurses do all this*, *what would be the role of the OT*? *If my role just covered activities like an OT*, *then that would be easy*. (*Charge Nurse*: *Unit 4203*)

*We*’*ve got to do our own job and then try to fit other people*’*s jobs in* (*staff Nurse*: *Unit 0902*)

*If they* [*the GetREAL team*] *stayed longer and all of these people* [*the unit staff*] *stayed out of the office*.., *but these people now have all gone back to their own little positions*, *and us little people are still left on the floor trying to muddle around to get it right and it is hard for us*. (*Nursing Assistant*: *Unit 3301*)

#### Leadership

Sustainability also required leadership committed to the aims of the intervention. This included the role of the Link Person on each unit as well as the support from the unit manager.

*When* [*the intervention team*] *came in*, *they motivated the staff*, *without them*…*we needed someone to take leadership of that. Nobody*’*s actually took over where they left off. Because I mean*, *each and every one of us*, *have got enough on our job role as it is* (*Deputy Manager*: *Unit 3301*)

It appeared that some members of staff nominated as a Link Person were not truly “signed up” to continue to champion the intervention long term. Some units were not aware of who their nominated person was, or the Link Person was nominated by management for the role rather than volunteering. Some viewed the role of Link Person as an additional burden without reward and those assigned to it may not have been best placed to maintain the changes.

*They left us a box of tools didn*’*t they and a nice file with all the activities planned and where we could go and where we could find them and how to*, *you know do them sort of thing. There are files for that in the office. Is it still in the office*? (*Nursing Assistant*: *Unit 4204*)

*Well we got volunteered didn*’*t we*, *I mean*, *the OT and I ended up leading it and we were saying* - *oh when did we volunteer for that then*? - *we didn*’*t know that we were leading it did we until this came* [*the Action Plan*] *and it was like*, *by who*? *and it was oh*, *oh it*’*s us*? (*Charge Nurse*: *Unit 4203*)

As one unit manager indicated, there had been “some slippage” since the GetREAL teams left and spoke of taking her “eye off the ball”. It was also noted that the staffing required to deliver rehabilitation were not adequately valued within the organisation and, consequently, staff with specific skills to facilitate activities were sometimes moved to other units.

*The OTs were coming and going*, *there was no activity coordinator*, *we had one*, *it got taken*, *I mean the management of this place in terms of patients*’ *activities and groups and stuff was literally nil*. (*Senior Nurse*: *Unit 0804*)

*The activity coordinator started quite a lot of things rolling you know*, *but he couldn*’*t continue because they* [*management*] *had to move him somewhere else*, *you know and the same thing happened with the OT as well* (*Staff Nurse*: *Unit 0804*)

#### Competing priorities

Patient safety and record keeping are essential elements of health service provision but within the rehabilitation units in this study such tasks seemed to compete for staff time with the facilitation of activities for patients. In the absence of the ‘permission’ provided by the intervention team, it appeared that staff anxieties about completing paperwork were prioritised.

*You wouldn*’*t get pulled up on what activities you*’*re not doing*, *you get pulled up on your paperwork that*’*s not filled in*, *or you haven*’*t ticked these boxes*. (*Staff Nurse A*: *Unit 3106*)

*We are the primary nurses of some of these residents*, *we*’*re the ones that are feeding back to MDT about their activities*, *trying to work with people to get them moved on*, *but we are not spending that time doing these activities with people. We get to do all the day*-*to*-*day*, *boring stuff*; *the mandatory stuff that needs doing*, *you have to do that*. (*Staff Nurse B*: *Unit 3106*)

## Discussion

This qualitative study aimed to provide an understanding of the strengths and weaknesses of the GetREAL intervention that may have affected its efficacy in facilitating patient engagement in activities [5]. Our findings revealed that although the intervention was positively received by staff in the rehabilitation units, the skills gained and changes to the unit’s processes and structures that were agreed with the intervention team were not sustained after they left. Previous studies have shown that a positive attitude towards or belief in an intervention alone is not sufficient for it to be maintained [9]. The process of implementation must be active [9] and thus we may have underestimated both the time needed to embed new approaches [10] and the responsibilities of the Link Person. For instance the Link Person’s role in supporting ongoing change in practice may have needed to be more strongly formalised, particularly in public sector settings such as the NHS where agents of change often have less power to exercise their discretion than in other settings [11].

Overall, the main factors associated with the lack of sustainability of the intervention appeared to be systemic issues, that is, those related to NHS mental health services as a whole, rather than located in discrete elements of the intervention. Within the NHS, systemic dynamics operate between patients and professionals, between different professionals within teams, and between clinicians and management [12]. Beyond these, implementation must more fully accommodate the organisational, economic and political contexts [13]. Among these different constituencies, dissonant goals and values tend to create tensions that are not easily resolved [14]. As May and colleagues suggest, implementation failures are too often attributed to the behavioral inertia of individual professionals, rather than socio-organisational factors [15]. Thus, implementation strategies may be overly focused on front-line professionals with insufficient buy-in from senior managers and commissioners [16].

Studies repeatedly demonstrate that change is difficult to sustain in organisations. Chapman [17] states that changes introduced in one element of an organisation often fail because that change is governed by the existing elements of the organisational system which create a tendency for the system to revert to its original state. Despite this there are certain factors that have been identified that can make a difference. Torrey and colleagues found that “active, engaged and visible leadership” strongly influenced the successful implementation of an intervention [9]. They argue that there will always be barriers in any organisation, but it is strong leaders who actively work around the barriers they encounter that make the difference to creating and sustaining change [9]. Furthermore, it is important that changes in practice are supported by changes in the flow of work processes [18] so that changes can be made routine. Unfortunately while attempts were made during the intervention to get leadership involved and put processes in place at the unit level, these were not fully realised.

It was difficult to anticipate one of the major external threats to the implementation of our intervention. During this study, the economic recession led to resource cuts, staff reshuffling, redundancies, pressure to maintain patient turnover, and the threat of closure of services. It is likely that this had multiple effects on the sustainability of the intervention. Primarily sufficient resources are crucial to support the process of change [11] and secondly staff turnover is a known hindrance to implementation of evidence- based interventions, particularly in mental health settings [19]. Economic pressures may have also impacted on the capacity for staff and management to fully engage with the aims of the intervention in terms of sustained change in practice. It seems likely that some staff effectively declined an investment in service change [14]. For instance, when visiting one of the units to conduct a focus group with unit staff who had received the GetREAL intervention (just four months earlier) we arrived to find that staff had been informed that morning that their unit was closing. In this context, morale-building and sustaining an ethos of recovery-oriented practice seems improbable.

There also appeared to be resistance amongst staff to relaxing role boundaries, which may have effected sustainability of the intervention [20]. While staff seemed able to broaden their responsibilities with encouragement and permission from the intervention team, some staff expressed strongly held views about the remit of their role. While resistance can be overcome by empowering staff to make the change themselves, [21] attempts to give staff ownership for driving the change in this intervention (through collaborative working, agreeing plans of action and establishing link persons) was not embraced.

It has long been known that a key to sustained change in the NHS requires explicit analysis of roles, structure and culture and how these are integrated across all aspects of the work [12]. However, as recent scandals involving the existence of unacceptable conditions of care in inpatient settings have highlighted [22], these aspects are often overlooked due to a focus on achieving targets rather than delivering high quality patient focussed care. The difficulty staff had in maintaining changes in their practice after the intervention team left appears to have been driven by a lack of support from senior management, competing priorities for resources and the organisation’s imperative to prioritise externally imposed targets [22, 23]. In other words, the culture of support for staff to maintain change was absent and staff had little choice but to revert to previous practice [14].

### Limitations

Although we attempted to capture the full diversity and range of inpatient mental health rehabilitation units that participated in the GetREAL intervention, the settings, experiences and perspectives may not reflect those of other rehabilitation units in the UK. While the sampling method used in this study (maximum variation sampling to include only half of the intervention sites) is a standard qualitative method, it must be acknowledged that not using random selection introduces a potential source of bias that may have effected the representativeness of the sample. Additionally the focus groups were carried out between 2 and 9 months post intervention, which may have produced a degree of recall bias. Furthermore, the emphasis within our focus group discussions may have changed over time. For instance, groups conducted early on may reflect the enthusiasm of a “honeymoon” period, whereas later group discussions may reflect some of the developing barriers. Additionally, whilst every effort was made to arrange a convenient time for the focus group when as many staff who had participated in the intervention were present, not all staff could attend. This may have meant that some important aspects of staff experience were therefore unable to be captured.

Power relationships are an important consideration within the conduct of focus groups and the presence of senior members of staff may have influenced open disclosure by participants. However, the interviews were directed towards the intervention rather than a critical investigation of staff dynamics; the difficulties of the latter tended to emerge obliquely and we saw little sign of staff reticence in the discussion of problematic issues. A further limitation may be the absence of other stakeholders such as senior management staff and service commissioners who may have been able to provide an understanding of some of the external threats to interventions such as ours.

## Conclusion

This study has illustrated some of the inter-related factors which operate at different levels within and outside of NHS organisations and impact on success of complex interventions. These factors need to be considered when designing interventions to ensure adequate buy-in from senior staff. In addition, specific strategies that reinforced the aims of our intervention over time may have improved its efficacy [24]. The results of our main trial showed that the intervention was not effective at increasing service users’ engagement in activities at 12 month follow-up [5]. Our study suggests that the weakest part of the intervention was the Reinforcing (sustaining) phase and therefore it may be that strengthening of this aspect would improve its effectiveness. However, we fully acknowledge that this cannot be concluded from our study findings and a further trial that addressed this aspect of the intervention would be required.

## Appendix

**Table 2:** Coding tree/analytical framework

Category	Theme	Data (Codes)
The challenges of working in rehabilitation units	1. The complex nature of the client group	- Patients’ relationships with staff
		- Difficulties engaging patients
	2. Impact of inappropriate referrals of patients prematurely transferred or with longer term needs	- Difficult to motivate patients
		- Lack of support for staff
		- Referrals of chronic patients
	3. Inadequate rehabilitation-focused leadership and staffing	- Lack of formal structures
		- Management
		- Resources
		- Workload
		- Staff held views on activity
Impact of the GetREAL intervention	1. Planning and reflection	- Provided structure and direction
		- Time management
		- Coordinate and organise activities
	2. External and alternative perspectives	- Taking interest in patients
		- Insight into patients
		- Interaction with patients
		- New ideas, tools and practices
	3. Dissolving role boundaries	- Changed work practices
		- Culture of rehabilitation
		- Flexibility
		- attitudes
	4. Collective working and responsibility	- Team working
		- Shared responsibility
		- Change in ward atmosphere
	5. Motivation	- Staff confidence
		- Accountability
		- Prioritising activity
Barriers to Sustainability	Intervention-related:	- lack of information
	1. Insufficient preparation	- practicalities
	2. Brevity of engagement with the intervention team	- Length of intervention
		- Top-up session
		- Lack of follow up
	3. Misunderstanding of the aims of the intervention	- Accountability
		- Staff focused versus patient focused intervention
		- Not sustaining changed practices
	Contextual issues:	- Workload
	4. resources	- Staffing
	5. Role boundaries	- management
		- rigidity
		- Permission
		- Defending roles and responsibilities
	6. Leadership	- Lack of rehabilitation focus
		- Paperwork driven
		- Lack of formal structures
	7. Competing priorities	- Low importance of activity in organisation
		- External targets

Note: Codes overlap between themes and are inter-related

## References

[1] Joint Commissioning Panel for Mental Health (2012). Guidance for commissioners of rehabilitation services for people with complex mental health needs.

[2] Macpherson R (2004). Supported accommodation for people with severe mental illness: a review. Adv Psychiatr Treat.

[3] Curson DA, Pantelis C, Ward J, Barnes TR (1992). Institutionalism and schizophrenia 30 years on. Clinical poverty and the social environment in three British mental hospitals in 1960 compared with a fourth in 1990. Br J Psychiatry.

[4] South London and Maudsley NHS Trust: Patient social engagement and attendance at organised activities on acute psychiatric wards within the South London and Maudsley Trust: An observational study and audit of 16 acute wards. Trust Report, 2004.

[5] Killaspy H, Marston L, Green N (2015). Cluster randomised controlled trial to assess the clinical effectiveness of a staff training intervention in inpatient mental health rehabilitation units in increasing patients’ engagement in activities. Lancet Psychiatry:.

[6] Killaspy H, Cook S, Mundy T (2013). Study protocol: cluster randomised controlled trial to assess the clinical and cost effectiveness of a staff training intervention in inpatient mental health rehabilitation units in increasing service users' engagement in activities. BMC Psychiatry.

[7] Archer M, Bhaskar R, Collier A, Lawson T, Norrie A (1998). Critical Realism: Essential Readings.

[8] Shediac-Rizkallah MC, Bone LR (1998). Planning for the sustainability of community-based health programs: conceptual frameworks and future directions for research, practice and policy. Health Ed Res.

[9] Torrey WC, Bond GR, McHugo GJ, Swain K (2012). Evidence-based practice implementation in community mental health settings: The relative importance of key domains of implementation activity. Adm Policy Ment Health.

[10] Birkmann JC, Sperduto JS, Smith RC, Gill KJ (2006). A collaborative rehabilitation approach to the improvement of inpatient treatment for persons with a psychiatric disability. Psychiatr Rehabil J.

[11] Fernandez R, Rainey HG (2006). Managing Successful Organizational Change in the Public Sector. Public Adm Rev.

[12] Menzies-Lyth I: A psychoanalytic perspective on social institutions. In: E. Bott Spillius, ed. Melanie Klein Today. Developments in Theory and Practice, Volume 2: Mainly Practice. London: Routledge; 1988: 284–299

[13] Tansella M, Thornicroft G (2009). Implementation science: understanding the translation of evidence into practice. Br J Psychiatry.

[14] Bray J (1999). An ethnographic study of psychiatric nursing. J Psych Mental Health Nurs.

[15] May C, Finch T, Mair F (2007). Understanding the implementation of complex interventions in health care: the normalization process model. BMC Health Services Research.

[16] Redfern S, Christian S, Norman I (2003). Evaluating change in health care practice: lessons from three studies. J Eval Clin Prac.

[17] Chapman J (2004). Systems Failure 2nd Ed.

[18] Rapp CA, Goscha RJ, Carlson LS (2010). Evidence-based practice implementation in Kansas. Community Ment Hlt J.

[19] Woltman EM, Whitley R, McHugo GJ (2008). The role of staff turnover in the implementation of evidence-based practices in mental health care. Psychiatr Serv.

[20] Oreg S (2006). Personality, context, and resistance to organizational change. Eur J Work Organ Psy.

[21] Poister TH, Streib G (1999). Performance measurement in municipal government: Assessing the state of practice. Public Admin Rev.

[22] Francis R: Report of the Mid Staffordshire NHS Foundation Trust Public Inquiry – Executive Summary 2013. London: The Stationery Office. Available at: www.midstaffspublicinquiry.com/report (accessed on 12 June 2014).

[23] The King’s Fund: Patient-centred leadership – rediscovering our purpose. Report from The King’s Fund Leadership Review 2013. London: The King’s Fund. Available at: http://www.kingsfund.org.uk/publications/patient-centred-leadership (accessed 12 June 2014).

[24] Berry K, Haddock G (2008). The implementation of the NICE guidelines for schizophrenia: Barriers to the implementation of psychological interventions and recommendations for the future. Psychol Psychother.

